# Better together: utilizing an interprofessional course and escape room to educate healthcare students about opioid use disorder

**DOI:** 10.1186/s12909-023-04899-6

**Published:** 2023-12-05

**Authors:** Kelsey K. Schmuhl, Steven Nagel, Ross Tamburro, T’Bony M. Jewell, Emily Gilbert, Anthony Gonzalez, Donald L. Sullivan, Jon E. Sprague

**Affiliations:** 1 The Ohio State University College of Pharmacy, Columbus, OH USA; 2 The Ohio State University College of Social Work, Columbus, OH USA; 3 The Ohio Attorney General’s Center for the Future of Forensic Sciences, Bowling Green State University, Bowling Green, OH USA; 4 Office of Ohio Attorney General Dave Yost & Bowling Green State University, Bowling Green, OH 43403 USA

**Keywords:** Interprofessional Education, Opioid Use Disorder, Escape Room

## Abstract

**Background:**

The aim of the present study was to determine the impact of an innovative interprofessional educational activity on healthcare professional students’ learning. The educational activity targeted student knowledge of opioid use disorder (OUD) and perceptions of working with an interprofessional team while caring for patients with OUD.

**Methods:**

Students from nursing, pharmacy, physician assistant, dentistry, social work, and medicine programs were recruited to participate in the interprofessional educational activity. The educational experience included seven asynchronous modules and a virtual synchronous escape room. Prior to the educational programming, participants completed a pre-survey that assessed their knowledge and attitudes towards working on an interprofessional team and perceptions of patients with OUD. The asynchronous modules were required in order to participate in the escape room and each module contained its own pre/post quiz to assess student knowledge.

**Results:**

A total of 402 students participated in the course. Prior to participating in the course, students disagreed that they had extensive educational experience with SUD (2.45 ± 0.79). The students displayed significant improvement in the knowledge based areas after completing the seven asynchronous modules. The largest significant area of knowledge-based improvement was seen in treatment of OUD where on the pre-quiz 65.54 ± 20.21% were answered correctly compared to 95.97 ± 9.61% on the post-quiz. Participation in the escape room significantly changed the students’ perceptions of working in interprofessional teams while managing patients with OUD. Of the eleven perception variables assessed, seven showed a significant increase in the post-survey. Following the escape room, participants also strongly agreed that they now would refer patients to colleagues in other disciplines.

**Conclusions:**

An interprofessional educational experience including both an asynchronous course and virtual synchronous escape room can increase participant knowledge around OUD and may improve student perceptions of working with an interprofessional team and caring for patients with OUD.

**Supplementary Information:**

The online version contains supplementary material available at 10.1186/s12909-023-04899-6.

## Introduction

Despite efforts to reduce the impact of the opioid crisis, the opioid epidemic has continued to surge [1]. In the United States, the most recent estimate is that more than six million individuals suffer from opioid use disorder (OUD) and 109,680 drug overdose deaths occurred in 2022 [2, 3]. During the first wave of the opioid epidemic from 1999 to 2010, prescription opioids were the primary driver of opioid overdose deaths [3]. The second wave (2010 to 2013) saw an increase in heroin related deaths and the third wave (2013 to present) has been associated with synthetic forms of opioids [3, 4]. A recent survey of the health professional schools in Ohio identified inconsistencies in the training of healthcare professionals in substance use disorder (SUD) and OUD [5]. There is, therefore, a critical need to enhance the interprofessional education of healthcare professionals on SUD and OUD [6]. Further, an interprofessional approach to training healthcare professional students about OUD and SUD has been suggested as the educational standard [7, 8]. Because healthcare professionals do not work in silos when providing care for patients with SUD, an interprofessional educational approach more closely resembles the true nature of the health care model [8].

A recent study of the pedagogical design of interprofessional escape rooms on knowledge recall and interprofessional skills demonstrated the positive impact an escape room design had on outcome measures [9, 10]. The escape room model has also been shown to facilitate communication and appreciation for an interprofessional approach to health care [11]. Furthermore, an in person escape room has been shown to improve pharmacy student knowledge with regards to SUD [12].

Ojeda et al., found that not all healthcare professionals with prescribing or dispensing authority were educated on how to screen for OUD, the association of adverse childhood experiences (ACEs) and SUD, the signs of SUD or how to use the prescription drug monitoring program (PDMP) system [5]. One novel aspect of this survey was that it included a focus on how healthcare students are trained on the relationship between ACEs and SUD. As patients that have experienced ACEs have a positive association for adulthood prescription opioid misuse [13] and are disproportionately represented in patients with SUD [14]. The aim of the present study was to determine the impact of an innovative interprofessional educational activity on healthcare professional students’ learning. The educational activity specifically targeted student knowledge of opioid use disorder (OUD) and student perceptions of working with an interprofessional team and caring for patients with OUD.

## Materials and methods

The Institutional Review Board (IRB) at The Ohio State University approved this study (protocol #2022E0900).

### Study design

The research design used a quasi-experimental pre/post-test survey design with no control groups. A total 402 health professional students from 13 programs representing eight universities in Ohio elected to participate in the course in the spring of 2023 and 379 students agreed to participate in the associated research study. A statewide approach was utilized in order to have the largest impact on healthcare professional training. These students were given the option to participate through their respective academic institutions’ required or elective courses that incorporated SUD in the curriculum. Prior to the educational programming, participants completed a pre-survey that assessed their attitude towards working on an interprofessional team and perceptions of SUD and patients with OUD. The questions regarding interprofessional teamwork were adapted from the Attitudes Toward Health Care Teams (ATHCT) scale [15] and a survey constructed to assess perceptions towards caring for patients with SUD and OUD [16]. The students then completed the 7-module asynchronous course around key concepts related to OUD: 1) Neurobiology of SUD; 2) Treatment of OUD Part 1; 3) Treatment of OUD Part 2; 4) ACEs; 5) Social Determinants of Health; 6) Motivational Interviewing; 7) Ethics and Stigma. The educational objectives from each of the asynchronous modules can be found in Supplemental Appendix. Each of the asynchronous modules were approximately 1 h in length and contained both a pre- and post-quiz in which the students had to receive at least an 80% on the post-quiz in order to “unlock” the next module. The first attempt on the post-quiz was used for assessment purposes.

In the synchronous interprofessional escape room, students worked in interprofessional teams to solve a complex patient case. The post-survey following the escape room contained the same knowledge and attitudes questions as the pre-survey and additionally included questions about intended behavioral changes as a result of the educational program. Pre- and post-quizzes and surveys were aggregated to protect participant anonymity.

### Asynchronous and synchronous module development

An educational committee was formed in May 2021 consisting of 32 faculty from healthcare programs from across Ohio. The committee represented medicine, pharmacy, nursing, dentistry, and physician assistant programs and was tasked with creating and implementing this educational experience. From this larger group, a subcommittee was formed which met monthly to work towards this goal. While the committee considered using an in-person, hybrid, or virtual event, it proceeded with a virtual model leveraging both asynchronous and synchronous components to increase accessibility of the program to students across Ohio. Once the format was decided, the committee partnered with an instructional design and educational technology team to create the learning material utilizing best practices in pedagogy and accessibility.

The asynchronous modules described above were housed in a Public-Facing learning management system (Canvas), which allowed students from many institutions to register for the course. Each asynchronous module followed a similar format which included an overview of the topic, pre-assessment, module content, and post-assessment. Content experts representing pharmacy, nursing, social work, and physician assistant programs collaborated to provide module content. These experts employed a variety of teaching methods in the asynchronous modules including recorded voice-over PowerPoint lectures, podcasts, guided readings, and a 360-degree interactive scenario which immersed students into the life of a person with SUD. The instructional design team reviewed each submitted learning material to ensure that relevant learning objectives and contextual information (e.g., overview text, priming questions) were included and embedded into the course template inside of Canvas for a simplified, consistent learning experience.

Following the completion of the asynchronous modules, students were required to attend a 3-h synchronous, virtual escape room hosted via Zoom in which they were assigned an interprofessional team to work through a complex patient case. To engage learners and encourage interprofessional collaboration, the educational committee implemented an escape room model for the patient case. Prior to completing the patient case, students were assigned to their interprofessional teams and placed into Zoom breakout rooms for 30 min to discuss their healthcare profession’s role in treating patients with SUD. Students were then given access to the patient case materials and allotted 90 min to complete the escape room activity.

The instructional design team deployed several specific technologies in the design of the escape room to create an engaging, interactive, accessible, and authentic learning experience. Most escape room activities consisted of linked Canvas pages to simulate branching pathways with additional interactive content housed in either H5P (e.g., quizzes or interactive videos) or Echo360 videos or interactive media embedded on the Canvas page. Additionally, Microsoft Forms with free response questions were embedded on activity pages to encourage interprofessional discussion and allow teams to provide their thoughts before progressing in the escape room activity.

The overall design of the escape room activities mimicked real-world scenarios. When the students first entered the escape room, they saw a patient in the emergency room who was brought by his brother for a suspected overdose. Through the successful completion of several activities where students practiced their skills related to motivational interviewing, medication reconciliation, interpreting clinical notes and reports from the PDMP, addressing social determinants of health, prescribing medications for OUD and withdrawal, and interpreting serum drug levels and vital signs, students received the final code to unlock the medication needed to save their patient’s life. During the escape room activities, facilitators from across disciplines and institutions rotated through breakout rooms to ensure learners were not stuck on any of the activities and that they were progressing sufficiently. Upon completion of the escape room, students met to debrief and reflect upon the experience.

### Data analysis

Pre- and Post-test and survey results were collected in Qualtrics. Data were analyzed in R 4.2.3 and the data was graphed with GraphPad InStat v.6.0 software. A 4-point Likert-type scale was used for survey responses whereas 1 = strongly disagree, 2 = disagree, 3 = agree, 4 = strongly agree. Likert-type scales are widely used in social science research and are usually treated as an interval level scale, but only if numbers are assigned to each potential survey response. By definition, response scales classified as interval level data are numerical, ordered with numbers assigned to each item on the scale. This gives the scale equal distance of measure between the response items, which by definition is interval level data. Therefore, the interval level data from Likert-type variables were compared using a paired t-test. Significance was established at *p* < 0.05 a priori.

## Results

### Demographic data

Of the 402 students enrolled in the course, the majority were nursing (53.7%) and pharmacy (35.1%) students with fewer students from physician assistant, dentistry, social work and medical programs (Table 1). Prior to participating in the course, students disagreed that they had extensive educational experience with SUD (2.45 ± 0.79). However, most agreed that they had previous experience working on interprofessional teams (3.29 ± 0.78).

**Table 1:** Demographic data of healthcare students participation

**Discipline**	N	
	(*N* = 402)	
Nursing	216	
Pharmacy	141	
Physician Assistant	25	
Dentistry	7	
Social Work	5	
Medicine	4	
Other	4	
**Previous SUD and IPE Experience**^**a**^		Mean ± SD
I have extensive educational experience with substance use disorder	284	2.45 ± 0.79
I have previous experience working on interprofessional teams	284	3.29 ± 0.78

^a^1 = strongly disagree, 2 = disagree, 3 = agree, 4 = strongly agree

### Asynchronous module knowledge assessment pre- and post-participation

**Fig. 1:**
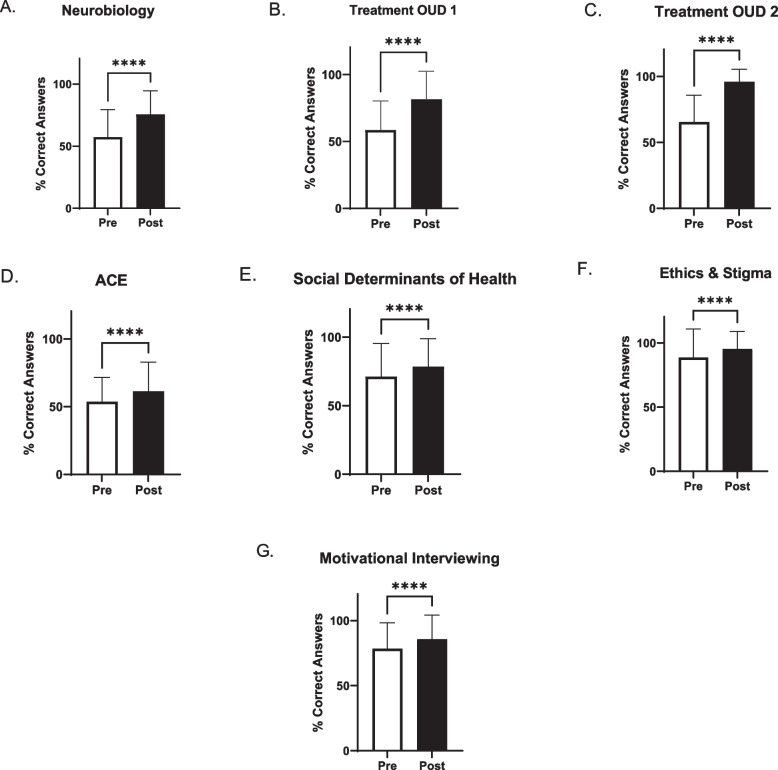
Asynchronous module knowledge assessment pre- and post-participation. Each value is the mean ± SD (*n* = 392–398). ****indicated a significant difference between pre and post module completion (*p* < 0.0001)

The students displayed significant improvement in the knowledge based areas after completing the seven asynchronous modules. Knowledge of treatment of OUD scores improved following the intervention (pre 65.54 ± 20.21% vs post 95.97 ± 9.61%, *p* < 0.0001). Although, students performed better on the post-quiz compared to the pre-quiz following the module on ACEs. However, only 61.37 ± 21.52% were able to answer the post-quiz questions correctly (Fig. 1).

### Escape room (synchronous module) pre- and post-participation student attitudes toward IPE and SUD and OUD perceptions

Participation in the escape room significantly changed the students’ attitudes toward working in interprofessional teams (Table 2). All 14 survey variables assessed pertaining to attitudes displayed a significant improvement in the post-survey. Participation in the program also significantly changed the students’ perceptions of working in interprofessional teams while managing patients. Of the eleven perception variables assessed, seven showed a significant increase in the post-survey findings compared the pre-survey (Table 3). Of the four perception variables that did not display significant changes in the post-survey, students strongly agreed that recovery from SUD is difficult, that peer support can have a positive impact on recovery and they disagreed with the statement that “patients who use methadone or buprenorphine to treat their OUD are not really in recovery.”

**Table 2:** Escape room (synchronous module) pre- and post-participation student attitudes to IPE^a^

**Attitudes toward IPE teams**	**Pre-** Mean ± SD	**Post-** Mean ± SD	***p* value**
Patients/clients receiving interprofessional care are more likely than others to be treated as whole persons	3.49 ± 0.64 (*N* = 379)	3.76 ± 0.58 (*N* = 320)	0.000001554*
Developing an interprofessional patient/client care plan is excessively time consuming	2.29 ± 0.80 (*N* = 379)	2.52 ± 1.06 (*N* = 320)	0.001044*
The give and take among team members helps them make better patient/client care decisions	3.51 ± 0.68 (*N* = 377)	3.76 ± 0.51 (*N* = 320)	0.00000005851*
The interprofessional approach makes the delivery of care more efficient	3.55 ± 0.61 (*N* = 377)	3.76 ± 0.53 (*N* = 320)	0.000002649*
Developing a patient/client care plan with other team members avoids errors in delivering care	3.53 ± 0.62 (*N* = 377)	3.73 ± 0.53 (*N* = 320)	0.000005015*
Working in an interprofessional manner unnecessarily complicates things most of the time	1.76 ± 0.89 (*N* = 377)	2.00 ± 1.21 (*N* = 320)	0.004343*
Working in an interprofessional environment keeps most health professionals enthusiastic and interested in their jobs	3.15 ± 0.63 (*N* = 377)	3.55 ± 0.59 (*N* = 320)	3.341e-16*
The interprofessional approach improves the quality of care to patients/clients	3.69 ± 0.52 (*N* = 377)	3.83 ± 0.45 (*N* = 319)	0.0005952*
In most instances, the time required for interprofessional consultations could be better spent in other ways	1.89 ± 0.87 (*N* = 377)	2.07 ± 1.16 (*N* = 319)	0.02154*
Health professionals working as teams are more responsive than others to the emotional and financial needs of patients/clients	3.34 ± 0.67 (*N* = 377)	3.66 ± 0.59 (*N* = 319)	2.17e-10 *
The interprofessional approach permits health professionals to meet the needs of family caregivers as well as patients	3.52 ± 0.60 (*N* = 376)	3.72 ± 0.55 (*N* = 319)	0.00004282*
Having to report observations to a team helps team members better understand the work of other health professionals	3.58 ± 0.57 (*N* = 377)	3.76 ± 0.50 (*N* = 319)	0.00003636*
Hospital patients who receive interprofessional team care are better prepared for discharge than other patients	3.61 ± 0.57 (*N* = 377)	3.77 ± 0.51 (*N* = 319)	0.00176*
Team meetings foster communication among team members from different professions or disciplines	3.60 ± 0.54 (*N* = 377)	3.81 ± 0.47 (*N* = 319)	0.000000124*

^*^Indicates significant difference between pre- and post- survey (*p* < 0.05)

^a^1 = strongly disagree, 2 = disagree, 3 = agree, 4 = strongly agree

**Table 3:** Escape room (synchronous module) pre- and post-participation student perceptions^a^

**SUD Perceptions**	**Pre-** Mean ± SD	**Post- ** Mean ± SD	***p* value **
Recovering from SUD is difficult	3.78 ± 0.49 (*N* = 372)	3.80±0.50 (*N*=355)	0.6517
Recovering from SUD is a lifelong process	3.72 ± 0.57 (*N* = 373)	3.82±0.50 (*N*=354)	0.03077*
More work needs to occur to minimize stigma related to SUD	3.77 ± 0.49 (*N* = 373)	3.85±0.44 (*N*=355)	0.02677*
Peer support can have a positive impact on chances of recovery	3.81 ± 0.45 (*N* = 373)	3.86±0.44 (*N*=355)	0.1525
Access to available treatment options for individuals in need is currently a problem	3.63 ± 0.57 (*N* = 372)	3.77±0.52 (*N*=355)	0.0006107*
Individuals with SUD have usually experienced significant adverse life events	3.42 ± 0.64 (*N* = 373)	3.71±0.52 (*N*=355)	2.2e-11*
Healthcare providers treat individuals with SUD differently than other patients	3.15 ± 0.67 (*N* = 373)	3.24±0.69 (*N*=355)	0.07815
Stigma among healthcare providers impacts the ability for patients with SUD to receive care	3.37 ± 0.69 (*N* = 372)	3.61±0.61 (*N*=355)	0.000001002*
It is important for individuals with OUD to have access to naloxone kits	3.69 ± 0.56 (*N* = 373)	3.82±0.47 (*N*=355)	0.001672*
Medications for OUD should be offered to all individuals with opioid use disorders	3.45 ± 0.67 (*N* = 373)	3.72±0.58 (*N*=355)	0.00000000746*
Patients who use methadone or buprenorphine to treat their OUD are not really in recovery	1.90 ± 0.97 (*N *= 373)	1.95±1.26 (*N*=355)	0.4397

^*^Indicates significant difference between pre- and post- survey (*p* < 0.05)

^a^1 = strongly disagree, 2 = disagree, 3 = agree, 4 = strongly agree

Following the escape room, students strongly agreed that their intentions (Table 4) were to change and work collaboratively on interprofessional teams when working with patients with SUD. Further, they strongly agreed that they will refer patients to colleagues in other disciplines and would be less likely to stigmatize patients with SUD.

**Table 4:** Students’ intention to change following the escape room (synchronous module)^a^

**Intention to change**	Mean ± SD
I intend to work collaboratively with interprofessional teams to care for patients at use for SUD	3.84 ± 0.45
I am more likely to refer my patients to colleagues in different disciplines	3.78 ± 0.49
I am less likely [stigmatize or make judgment upon] patients with SUD	3.83 ± 0.48
I better understand the challenges patients with SUD face when accessing the healthcare system	3.83 ± 0.48
I am confident in my ability to initiate discussions with my colleagues about OUD	3.80 ± 0.47

^a^1 = strongly disagree, 2 = disagree, 3 = agree, 4 = strongly agree

## Discussion

The results of this study indicate that a virtual interprofessional experience utilizing both asynchronous and synchronous components is an effective strategy to improve healthcare professional students’ knowledge and perceptions about working on interprofessional teams and caring for patients. The program described here highlights an innovative way to teach students about a particular healthcare issue and in this case, OUD.

Creating an educational experience that truly simulates interprofessional collaboration in a healthcare environment can be challenging for several reasons. One challenge is ensuring that many professions are represented on the team during the simulation. In this study, the majority of participants were from nursing and pharmacy programs. In Ohio, there are seven colleges of pharmacy which contributed to the increased number of pharmacy participants compared to other disciplines and one nursing program required the experience for their students, which increased the number of nursing students enrolled. While other programs (dentistry, physician assistant, social work, and medicine) were represented, it was to a lesser degree. When several pharmacy or nursing students had to be placed on a team, the researchers attempted to have several institutions represented when possible. This finding is similar to other interprofessional escape room research as many of them have been conducted with pharmacy and nursing audiences [9–12, 17]. One study aimed to prevent this problem by assigning roles to students when they started the escape room. Wettergreen et al. [10] had students randomly select from an envelope a label with a title of a health professional to role play. In the present study, students worked within their academic discipline which allowed students to work with professional healthcare colleagues while maintaining their own healthcare role.

Our findings of improved attitudes toward and working in interprofessional teams is consistent with recent evaluations of the use of escape rooms for interprofessional education. Interprofessional escape rooms have been shown to have a positive impact on the collaborative skills of health care students and that students report that escape rooms are a “fun way” to remove potential barriers [9]. Friedrich et al. [11] also reported that the use of an interprofessional escape room encouraged teamwork and the overall promotion of interprofessionalism.

This study also demonstrated that the asynchronous modules significantly improved participants’ knowledge. The educational committee felt strongly that all learners, regardless of profession, institution, or year in their program should enter the experience with similar foundational knowledge, and therefore the asynchronous course would be required before a student could participate in the escape room event. While one program described a 1-h asynchronous lesson the students completed prior to the escape room [9], to date, none have had the additional standardized asynchronous content that we required students to complete before the escape room event.

One essential element for the success of this program was the collaboration between the educational committee and instructional design team. This partnership made the creation of the virtual escape room possible as their team was able to leverage educational technology to make the activities engaging and interactive. The instructional design team further ensured all activities and educational content were digitally accessible for all learners. The instructional design team was available to help troubleshoot any logistical questions such as getting students enrolled in the course or technology problems that occurred during the escape room events. Throughout the development of the program, faculty met weekly with the instructional design team to discuss pedagogy, design activities, review digital accessibility considerations, and anticipate potential user experience or technical concerns for program learners. For asynchronous modules, the instructional design team designed a template module with consistent naming conventions, elements, and participation requirements. Additionally, the instructional design team worked with faculty to ensure that their learning materials were embedded in a consistent format reviewing for digital accessibility and captioned, as appropriate. For the synchronous activities, the instructional design team proposed potential technology solutions to enable various activities, storyboarded the flow of activities, developed and tested activities using a variety of e-authoring tools, and piloted those activities with their interns to ensure the activities functioned properly.

The multi-institutional nature of this program was also unique in that faculty and students from across Ohio (13 programs from eight different universities) were able to learn with and from each other. Begley et al. described the successful collaboration between a pharmacy program and physician assistant program at different institutions to conduct interprofessional telehealth cases [17], but other interprofessional escape room programs have been held within a single institution. Utilizing video conferencing platforms increases the feasibility of expanding this program to include healthcare students from across the country to engage in interprofessional education events.

The structure of the virtual escape room also proved to be beneficial for students. Taking time during the first 30 min to discuss logistics, allow students to build community with their team, and meet the facilitators for the event appeared to help students feel comfortable navigating the activity and resulted in minimal technology or logistics barriers while students worked through the escape room. After completion of the escape room, students were asked to debrief with their team and return to the large group for debriefing questions prior to leaving the event. In these sessions, students discussed what went well, what was most challenging, and provided constructive feedback.

A limitation of this study was the unequal representation of professions on the student interprofessional teams. Another potential limitation is that participants represented many levels of learners. Some were early in their professional program, while others were further along. While this allowed younger students to learn from older students and vice versa, some groups may have had different experiences depending on their team, which may have impacted their perceptions. Additionally, this study was a pre/posttest intervention study of an education intervention without a control group. Subsequently, we can only state that our students learned from our pedagogical design and not that our methods were any better or worse than other pedagogical designs.

## Conclusion

An interprofessional educational experience including both an asynchronous course and virtual synchronous escape room can increase participant knowledge and improves student perceptions of working with an interprofessional team and caring for patients. This model allows for standardization of content and increased accessibility for learners from many institutions and healthcare programs to learn with and from each other.

### Supplementary Information

**Additional file 1: Supplemental Appendix. **The educational objectives from each of the asynchronous modules.

## Data Availability

The datasets used and/or analyzed during the current study are available from the corresponding author upon request.
