# Supplementary material for: Better together: utilizing an interprofessional course and escape room to educate healthcare students about opioid use disorder
Source: BMC Med Educ. 2023 Dec 5;23:917. doi: 10.1186/s12909-023-04899-6 (PMC10696762; doi:10.1186/s12909-023-04899-6)
Supplement: Supplementary file 1 — Additional file 1: Supplemental Appendix. The educational objectives from each of the asynchronous modules. [file 12909_2023_4899_MOESM1_ESM.docx]

**Supplemental Appendix**: The educational objectives from each of the asynchronous modules.

**Module 1: Neurobiology of Substance Use Disorder**

1. Discuss the differences between euphoria, dysphoria, addiction and substance use disorder.
2. Recognize the DSM-5 criteria for substance use disorders
3. Identify based on pharmacological mechanism how individual classes of drugs regulate dopamine release in the nucleus accumbens
4. Discuss the cellular mechanisms that result in addiction-induced brain disease.
5. Identify the stages of the addiction cycle

**Module 2: Treatment of OUD 1**

1. Discuss relevant terminology and epidemiology of opioid use disorder (OUD)
2. Recognize harm reduction strategies related to OUD
3. Compare qualitative and quantitative urine drug screens and recognize their place in therapy
4. Summarize pharmacological treatment options for OUD, opioid overdose, and opioid withdrawal

**Module 3: Treatment of OUD 2**

1. Recognize the role of the American Society of Addiction Medicine (ASAM) in guiding treatment for patients with substance use disorders​
2. Examine the six dimensions of the ASAM assessment and how they assist in determining the appropriate level of care for the patient ​
3. Compare the differences between each level in the continuum of care​
4. Determine the appropriate level in the continuum of care based on specific patient criteria​
5. Discuss psychosocial interventions as it pertains to your patient's circumstances and desired goals.

**Module 4: Adverse Childhood Experiences (ACEs)**

1. Describe the rationale behind including ACEs score as part of a patient assessment
2. Identify when patients should be screened for ACEs
3. Name the ten factors that are included as part of the ACEs score
4. Describe the biological changes that occur during ACE exposures.
5. Understand the impact of ACEs in regards to epigenetics

**Module 5: Social Determinants of Health**

1. Identify six primary social determinants including the most common factors associated with each.
2. Understand how the social-ecological model is used to identify root causes of substance use.
3. Recognize the key social determinants associated with opiate misuse, opiate use disorder and overdose.
4. Discuss ways to reduce barriers to treatment access for individuals with opiate use disorder.
5. Use non-stigmatizing language to discuss issues related to substance use disorders.

**Module 6: Motivational Interviewing (MI)**

1. Describe the four key components of the Motivational Interviewing (MI) Spirit​
2. ​List the core skills used in MI​
3. ​Practice using the tools of MI to evoke change talk, and soften non-change talk​
4. ​Identify barriers to successful use of MI

**Module 7: Ethics and Stigma**

1. Identify terms to use and terms to avoid when speaking to people about addiction.
2. Recognize stigmatizing language in clinical interactions.
3. Explain the effects of stigmatizing language on persons with SUD.
4. Discuss communication strategies to facilitate positive interactions with persons with SUD.
5. Reflect on your own implicit biases related to persons with SUD.
